# CpG oligodeoxynucleotides inhibit the proliferation and osteoclastic differentiation of RAW264.7 cells†

**DOI:** 10.1039/c9ra11036d

**Published:** 2020-04-15

**Authors:** Yi Zheng, Wenwen Yu, Hongyan Li, Hongbing Lin, Zhen Chen, Huishan Chen, Peipei Zhang, Yue Tian, Xiaowei Xu, Yuqin Shen

**Affiliations:** Department of Periodontics, Hospital of Stomatology, Jilin University 1500 Qinghua Road Changchun 130021 Jilin China shenyqjlu@126.com; Key Laboratory of Organ Regeneration & Transplantation of the Ministry of Education, The First Hospital of Jilin University Changchun 130061 Jilin China; Department of Orthodontics, Hospital of Stomatology, Jilin University 1500 Qinghua Road Changchun 130021 China; Department of Orthodontics, Tianjin Stomatological Hospital, Nankai University Tianjin 300041 China

## Abstract

Clinical prevention and treatment of periodontitis-induced bone absorption remains a challenge. The anti-infection role of CpG oligodeoxynucleotides (CpG ODNs) is well known; however, their effect on osteoclasts is still unclear. Here, we show that some CpG ODNs can regulate osteoclastogenesis of RAW264.7 cells. The phosphorothioate CpG ODN was efficiently taken up by the cells within 1 h and distributed in the cytoplasm. BW006, YW001, YW002, and FC004 CpG ODNs significantly repressed cell proliferation by targeting several cyclin proteins to arrest the cells in the G2 phase and to further initiate cell apoptosis. Regarding differentiation, we selected six CpG ODNs (FC002, BW006, YW002, YW001, FC004, and MT01) that markedly inhibited the gene expression levels of *Nfatc*, *c-fos*, *RANK*, and *MMP9*. TRAP staining showed that only YW002, YW001, and FC004 suppressed osteoclast generation and maturation. These three CpG ODNs dramatically declined the protein levels of osteoclastogenic proteins by elevating the ratio of OPG/RANKL and also downregulating the inflammatory factors (TNF-α, IL-1β, IL-6, and IL-17) at different stages. Thus, the selected CpG ODNs may be a potential molecular therapy for the prevention and treatment of periodontitis-mediated bone resorption.

## Abstract 2

Clinical prevention and treatment of periodontitis-induced bone absorption remains a challenge.

## Graphical abstract

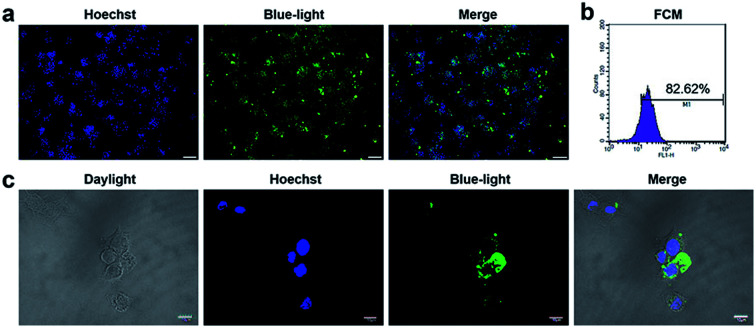

## Introduction

Periodontitis is a serious bacterial infection of the gums which could damage the soft tissue and destroy the bone that supports teeth.^1^ It is reported that periodontitis could affect up to 90% of the world's population.^2^ Epidemiological studies suggested that periodontitis could increase the risk of several diseases, such as rheumatoid arthritis, atherosclerosis and cancer.^1,3^ Therefore, prevention of periodontitis is important to improve general health.

*Porphyromonas gingivalis*, a Gram-negative anaerobic bacterium is the main etiologic agent for periodontitis.^4^ Bacterial DNA, as a special pathogen, could activate macrophages through toll like receptors (TLRs), and can trigger macrophages secret inflammatory cytokines.^5^ These inflammatory cytokines further induce the imbalance between osteoblasts and osteoclasts and lead to bone absorption.^6^

The main treating strategies for periodontitis are controlling inflammation and regenerating soft tissues.^7^ Inflammation can be effectively controlled but bone absorption is irreversible and requires bone tissue engineering to achieve regeneration. In recent clinical strategies, though various methods are applied, such as bone graft, guided tissue regeneration, growth factors, these still have inevitable weaknesses.^8^ There are rarely used methods that can prevent osteoclastogenesis.

Recently, more attention has been paid to the molecular therapy. TLRs are a class of immune molecules that closely associated with periodontitis. Previous studies indicated that unmethylated cytidine-phosphate-guanosine (CpG) DNA motif could also trigger a protective immune response that can enhance the host's ability to eliminate antigens.^9,10^ CpG sequences of bacterial DNA or synthetic CpG oligodeoxynucleotides (ODNs) can strongly initiate the immune system to resist infections.^10^ It was reported recently that CpG ODN has been widely used to resist tumor, infection, and allergy.^11–13^ Similarly, their application in prevention/reducing periodontitis through suppression of innate-like B cell apoptosis^14^ and promotion of protective Th1- and Th2-type cells^15^ has also been reported. Back to the 19th century, the researchers found that bacterial DNA injected into the joints could lead to arthritis and joint cartilage destruction.^16–18^ Then, Zou *et al.* reported the double regulation of CpG ODN during osteoclast differentiation, and he confirmed that the CpG ODN inhibited the osteoclast differentiation by repressing M-CSF to further decline RANKL in early osteoclast precursors but enhanced TNF-α and RANKL to strongly trigger RANKL-primed osteoclast differentiation.^19^ Moreover, the only CpG ODN but not oligonucleotide without CpG specially affected osteoclastogenesis.^20^ Interestingly, Krisher explained pathogen-induced osteoclastogenesis attributing to TLRs activation in RANKL-primed cells, but TLRs in early cells inhibited osteoclastogenesis.^21^ Their studies indicated that CpG ODN regulated osteoclastogenesis through binding with TLR9, just like Alla.^22^ Due to TLR9 dependent modulatory osteoclastogenesis, CpG ODN has been suggested as a potential immunotherapy for bone-degenerating diseases. However, the effect of CpG ODN on osteoclastogenesis is far from known.

In this study, in order to select effective CpG ODNs that can suppress osteoclastogenesis, we examined twelve ODNs for their effects on proliferation, apoptosis, osteoclastogenic differentiation, and release of inflammatory factors from macrophage of RAW264.7 cells. We hope to identify a CpG ODNs with significant anti-infection activity for the prevention and treatment of periodontitis-induced bone absorption.

## Results and discussion

### Effective uptake of ODN by RAW264.7 cells

**Fig. 1:**
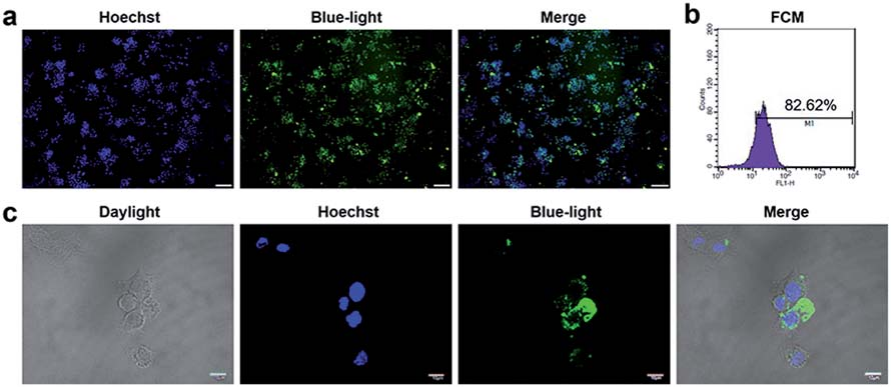
The uptake of CpG ODN on RAW264.7 cells. (a) Microscopic images of FAM-ODN-positive RAW264.7 cells under ultraviolet light, blue light and merged of both. Scale bar: 100 μm. *n* = 3. (b) Flow cytometry analysis of FAM-ODN-positive RAW264.7 cells. *n* = 3. (c) Laser co-focusing detection of location of FAM- ODN on MC3T3 cells. From left to right, the stained cells were detected by daylight, ultraviolet light, blue light and merged of three. *n* = 3.

CpG ODN has been widely used in the research on immunology and cancer. However, the poor stability and low intracellular uptake limit its clinical application.^23^ To overcome these problems, ODN was chemically modified. Phosphorothioate modification of ODN is a common strategy to achieve more stability against enzymatic degradation.^24–26^ Phosphorothioate has been found to reduce the activity of a variety of extra and intracellular nucleases.^27^ Phosphorothioate ODN was found effective in creating ODNs for antisense knockdowns.^28^ Phosphorothioate ODNs downregulate gene expression by hybridizing to a target mRNA, which in turn either inhibits mRNA maturation, enables RNase H-mediated degradation of the transcript, or blocks translation.^29^ To detect the uptake and distribution of ODN in RAW264.7 cells, we conducted fluorescence microscopy and laser co-focusing detection. The results show that the phosphorothioate ODN could quickly enter the RAW264.7 cells within 1 h. The FAM-ODN positive cells were estimated to be more than 80% by fluorescence microscopy (Fig. 1a) as well as the FCM assay (quantity of transfection efficiency was found to be 82%) (Fig. 1b). The high magnification images exhibit that ODN was mainly located in the cytoplasm and did not enter the nucleus (Fig. 1c). Thus, phosphorothioate ODN penetrated cells quickly and efficiently without vehicle and were mainly distributed in the cytoplasm. In previous reports, ODN entered the cells by endocytosis, and initiated signal transduction cascades through TLR9.^30^ It is reported that MT01 ODN was taken up by MG63 cells within 12 h.^31^ In our study, CpG ODN uptake peaked at 1 h in RAW264.7 cells. Considering the immune response, Zhang and Zheng achieved the delivery of CpG ODN and MT01 ODN using graphene oxide-chitosan nanocomposites and *N*-isopropylacrylamide-modified polyethylenimine (PEN).^32,33^ Furthermore, CpG ODN was localized in the cytoplasm of the cell and did not enter the nucleus, which was similar to the results of Hou and Zhang.^31,32^ This was in accordance with the localization of TLR9 in the endo-lysosome.^30^ Therefore, the phosphorothioate CpG ODN not only rapidly and efficiently entered the cells, but also accumulated in the cytoplasm.

### CpG ODNs suppressed proliferation and promoted apoptosis of RAW264.7 cells

During periodontitis, an increase in the levels of inflammatory cytokines, such as IL-6, TNF-α, and IL-1 can stimulate the collection of mononuclear macrophages and immune cells to initiate the osteoclast differentiation of the osteoclast precursor cells, and the mature osteoclasts are able to absorb alveolar bone tissue.^34^ Here, we first applied RAW264.7 cells as a model to examine the influence of ODN on the proliferation of osteoclast precursor cells. We employed 2006 as the positive control and PBS as the vehicle control and compared with the experimental ODNs.

**Fig. 2:**
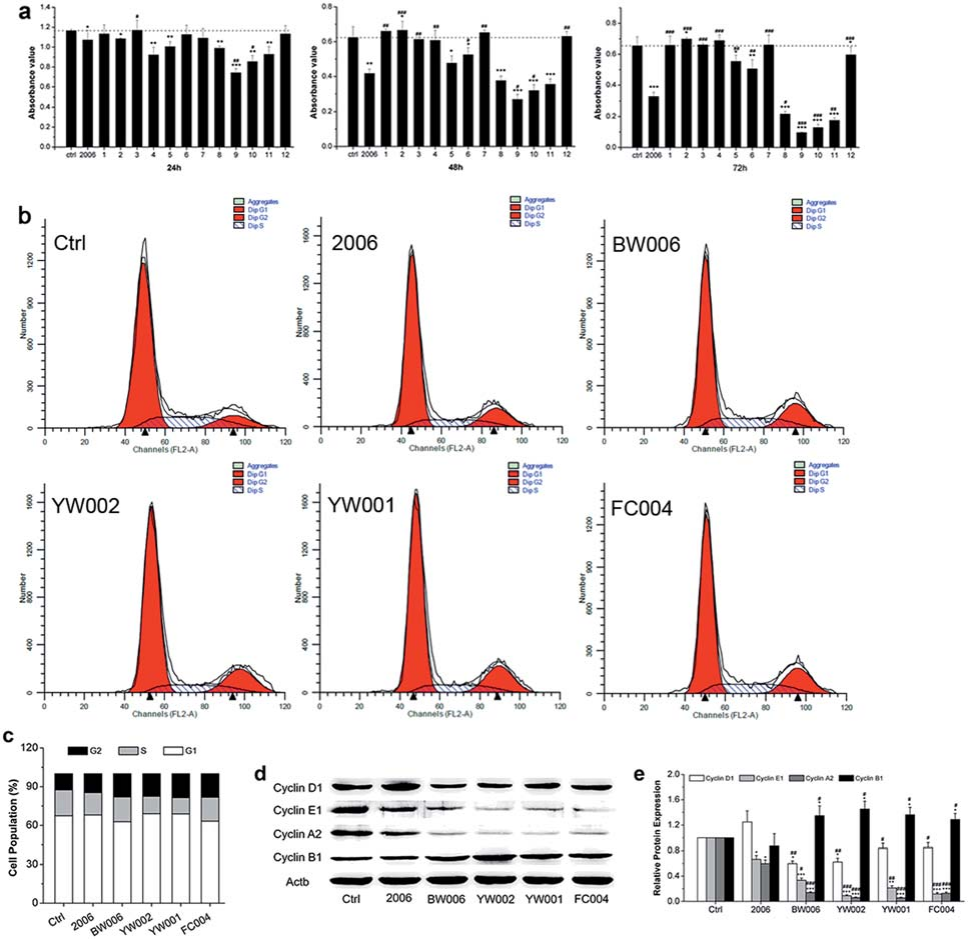
Cell proliferation analysis. (a) The cell viability test of RAW264.7 cells co-cultured with twelve ODNs for 24 h, 48 h and 72 h by MTT assay. *n* = 6. (b) Cell cycle examination by FCM after co-culture for 48 hours. (c) Statistical analysis of cell population according to (b). *n* = 3. (d and e) Western blot analysis (quality in (d) and quantity in (e)) of cyclin proteins of RAW264.7 cells transfected by CpG ODNs for 48 hours. *n* = 3. Data represent the mean ± SD of three independent experiments. *P < 0.05 *vs.* the control group and ^#^*P* < 0.05 *vs.* the 2006 group by paired *t*-test.

For detection of the effect of ODNs on cell proliferation, we performed MTT assay. Incubation for 24, 48, and 72 h revealed that most ODNs had a small effect on the proliferation of RAW264.7 cells except BW001, FC001, BW006, YW002, YW001, and FC004. These six CpG ODNs markedly and gradually suppressed the cell proliferation with extended treatment times, particularly BW006, YW002, YW001, and FC004, which lowered cell proliferation more than 2006 CpG ODN (Fig. 2a).

To investigate the underlying mechanism of inhibition of cell proliferation, we examined the cell cycle and cycle-related proteins. After co-culturing with 2006, BW006, YW002, YW001, and FC004 for 48 h, the results of cell cycle revealed that BW006 and FC004 mainly showed declined cell percentage of G1 phase, YW002 and YW001 mainly downregulated S phase population, but they all greatly increased the cell percentage of G2 phase, as compared with both PBS and 2006 group (Fig. 2b and c). Meanwhile, the cyclin proteins (D1, E1, A2, and B1) were affected by ODNs, and cyclin D1, E1, A2 presented significant reduction, while cyclin B1 was dramatically augmented.

Regarding the effect of CpG ODN on cell proliferation, we selected four effective CpG ODNs (BW006, YW002, YW001, and FC004) that significantly affected the fate of the osteoclast precursors. The cell cycle was regulated by cyclic proteins and cyclic protein dependent kinase complex. Among them, cyclin A, cyclin D, and cyclin E proteins mainly promoted eukaryotic cells from the G1 phase into the S phase. Cyclin B decides whether the cells should pass the DNA damage checkpoint of G2/M phase. The expression of cyclin B reached its peak during M phase, then got degraded after the cells had passed through the M phase.^35^ In our results, BW006, YW002, YW001, and FC004 CpG ODNs apparently suppressed the expression of cyclin A, cyclin D, and cyclin E proteins, which led to a decreased cell population in G1 and S phases. The dramatically elevated levels of cyclin B in response to the above four CpG ODNs indicated that most cells were blocked in the G2 phase, while the subsequent cell apoptosis assay proved that these CpG ODNs promoted cell apoptosis.

**Fig. 3:**
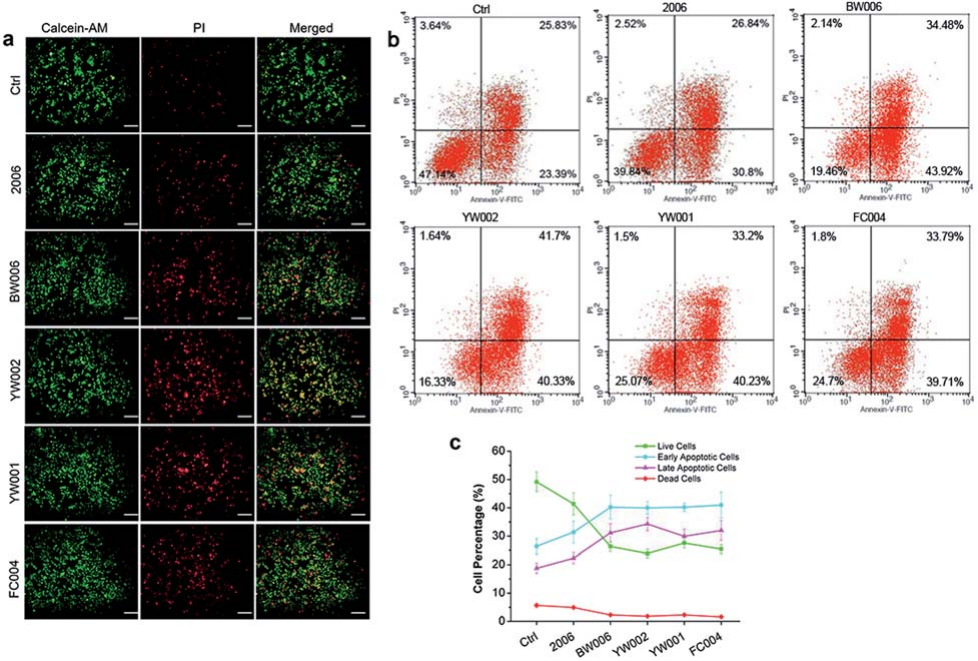
Cell apoptosis analysis. (a) The live-dead cell staining of RAW264.7 cells co-cultured with twelve ODNs for 72 hours. Scale bar: 100 μm. *n* = 3. (b) Cell apoptosis examination by FCM after co-culture for 72 hours. (c) Statistical analysis of cell population according to (b). *n* = 3. Data represent the mean ± SD of three independent experiments. *P < 0.05 *vs.* the control group and ^#^*P* < 0.05 *vs.* the 2006 group by paired *t*-test.

The elevated G2 phase indicated to test the cell apoptosis. The live-dead cell assay showed a higher percentage of dead cells (red cells) in BW006, YW002, YW001, and FC004 groups than PBS and 2006 groups (Fig. 3a). Cell apoptosis assay revealed these dead cells (red cells) from both early and late apoptosis, and the live cell population was also declined (Fig. 3b and c).

Thus, BW006, YW002, YW001, and FC004 CpG ODNs affected the expression of cyclin proteins to suppress G1 and S phases and block RAW264.7 cells in G2 phase, inducing cell apoptosis and decreasing cell proliferation.

### CpG ODNs inhibit osteoclast differentiation of RAW264.7 cells by elevating the OPG/RANKL ratio

**Fig. 4:**
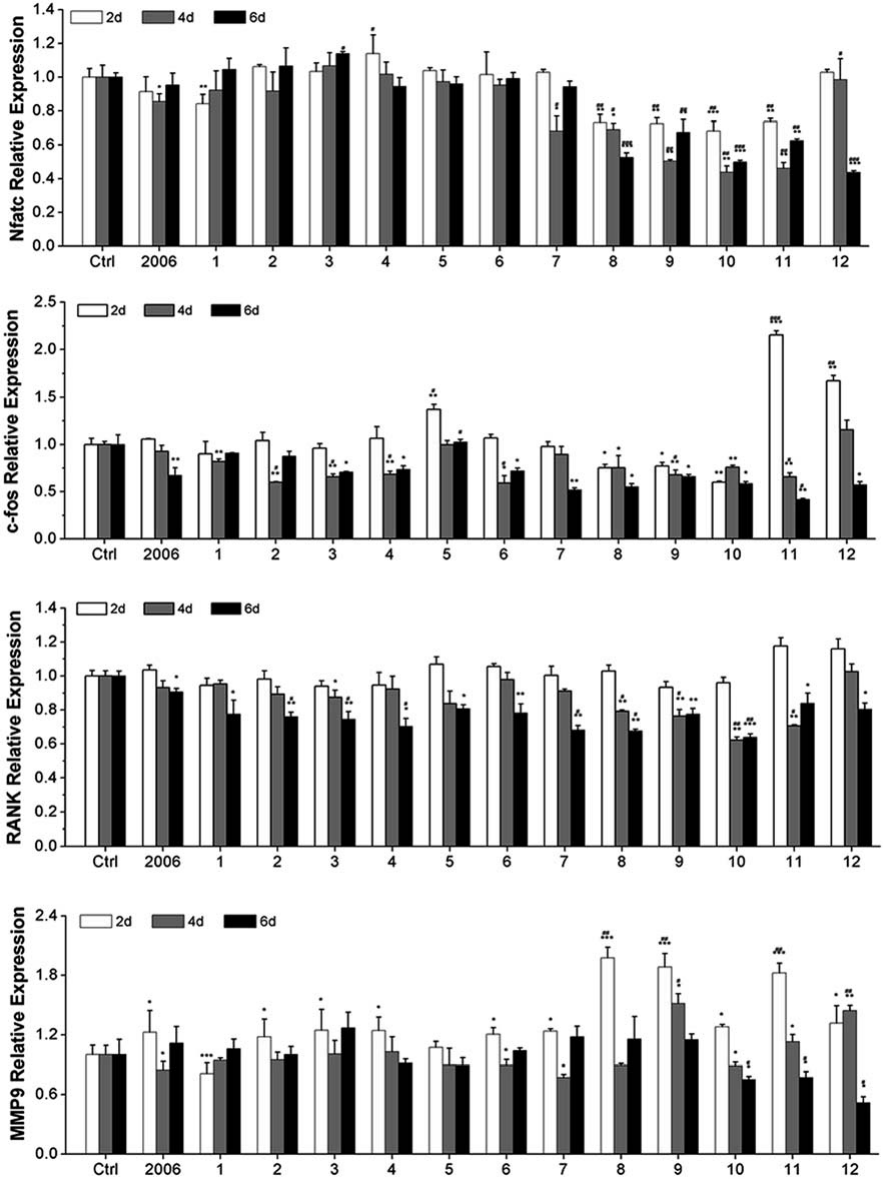
Osteoclast differentiated genes detection. The gene level of *Nfatc*, *c-fos*, *RANK* and *MMP9* after co-culture with ODNs for 2, 4 and 6 days were examined by qRT-PCR. *n* = 3. Data represent the mean ± SD of three independent experiments. *P < 0.05 *vs.* the control group and ^#^*P* < 0.05 *vs.* the 2006 group by paired *t*-test.

**Fig. 5:**
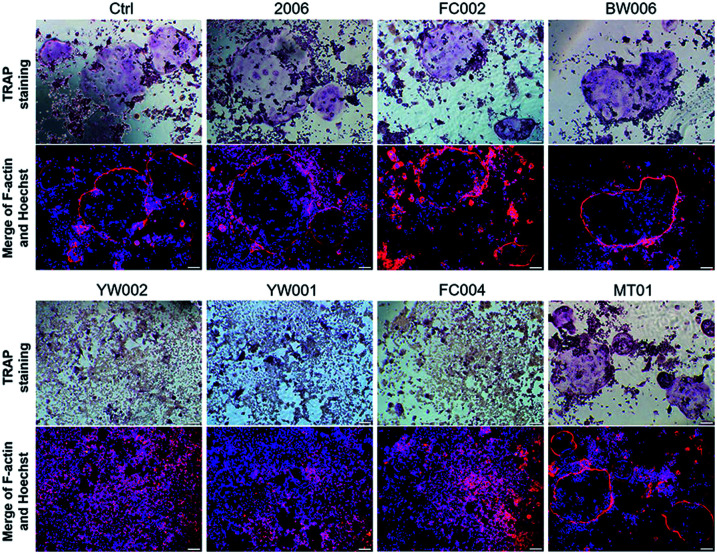
Mature osteoclast detection. The TRAP^+^, F-actin ring and multinuclear cells were detected after co-culture with ODNs for 4 days by TRAP staining, rhodamine phalloidin staining and Hoechst staining, respectively. *n* = 3.

**Fig. 6:**
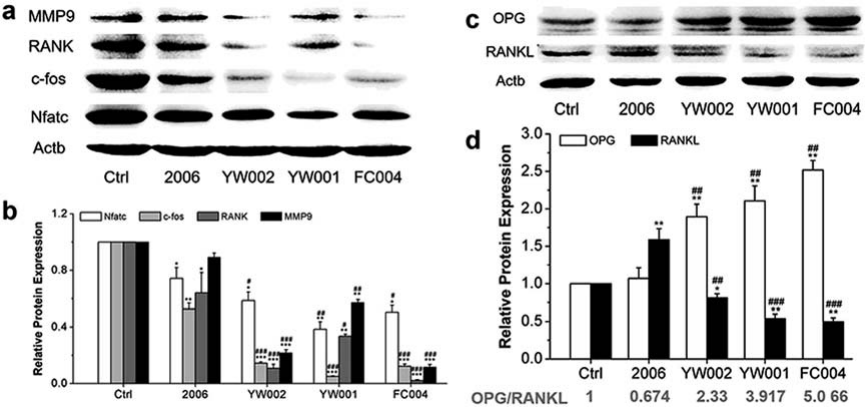
Osteoclast differentiated proteins detection. Western blot analysis (quality in (a), (c) and quantity in (b), (d)) of Nfatc, c-fos, RANK and MMP9 as well as OPG and RANKL proteins after co-culture with ODNs for 7 days. *n* = 3. Data represent the mean ± SD of three independent experiments. *P < 0.05 *vs.* the control group and ^#^*P* < 0.05 *vs.* the 2006 group by paired *t*-test.

Zou *et al.* thought that CpG ODN induced the osteoclast differentiation of RANKL-primed pre-osteoclast cells with increasing concentration and incubation time.^19^ Alla found that CpG ODN could transform the sustained phosphorylation of ERK to transient phosphorylation to decrease c-fos levels, which inhibited osteoclast differentiation.^36^ Next, we examined the effects of ODN on the differentiation of osteoclast precursor cells, RAW264.7 cells. According to the preliminary selection by qRT-PCR assay, the FC002, BW006, YW002, YW001, FC004, and MT01 ODNs mainly reduced the expression of *Nfatc*, *c-fos*, *RANK*, and *MMP9* on day 6 (Fig. 4). *Nfatc*, *c-fos*, and *RANK* are the main genes for osteoclast differentiation, and *MMP9* is the main functional gene of the osteoclasts. The selected six ODNs were used for the TRAP assay. TRAP is a unique marker enzyme of the osteoclasts and is widely used as a positive test index for osteoclast differentiation.^37^ In the presence of RANKL, RAW264.7 cells gradually fused to become big cells containing multiple nuclei, and these big cells were stained positive (purple) by TRAP staining, and showed complete F-actin ring through rhodamine phalloidin staining (Fig. 5). These data indicated that RAW264.7 cells differentiated into mature osteoclasts. In the groups of PBS, 2006, FC002, BW006, and MT01, there were more big multiple nuclei and TRAP positive cells, while these cells were not frequent in the groups of YW002, YW001, and FC004, which, nevertheless, showed bits of fusion cells that were smaller without complete F-actin ring. Therefore, YW002, YW001, and FC004 CpG ODN significantly suppressed the osteoclast differentiation of RAW264.7 cells. Western blot showed reduced expression of Nfatc, c-fos, RANK, and MMP9 proteins induced by YW002, YW001, and FC004 CpG ODN (Fig. 6a and b). In the process of osteoclast differentiation, OPG/RANKL/RANK is always the ultimate regulation mechanism.^38^ In our results, we have detected the upregulated OPG and downregulated RANKL levels in YW002, YW001, and FC004 groups as compared with PBS and 2006 groups, with an elevated ratio of OPG/RANKL (Fig. 6c and d).

Therefore, YW002, YW001, and FC004 suppressed the osteoclast differentiation of RAW264.7 cells through the OPG/RANKL/RANK pathway.

### The CpG ODNs influenced the secretion of inflammatory factors in RAW264.7 cells

**Fig. 7:**
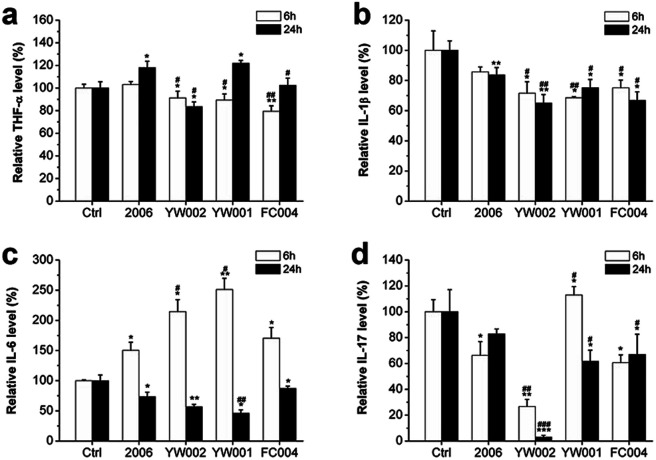
Inflammatory factors detection. The secretion of TNF-α (a), IL-1β (b), IL-6 (c) and IL-17 (d) were tested by ELISA assay after co-culture with ODNs for 6 and 24 hours. *n* = 3. Data represent the mean ± SD of three independent experiments. *P < 0.05 *vs.* the control group and ^#^*P* < 0.05 *vs.* the 2006 group by paired *t*-test.

The recent reports assumed that bacterial plaque was the promoter of periodontitis. Bacteria, their endotoxins, and metabolites activated the periodontal inflammatory immune response and initiated the immune cells to produce a large number of inflammatory factors, such as TNF-a, IL-1, and IL-6. These inflammatory cytokines could promote osteoclast differentiation to lead to the absorption and destruction of alveolar bone.^1,39–42^ The DNA from *Actinobacillus actinomycetemcomitan*, *Porphyromonas gingivalis*, and *Peptostreptococcus micros* could induce the release of TNF-α and IL-1 from gingival fibroblasts; therefore, TNF-α and IL-1 were considered the key factors in alveolar bone absorption.^43^ The cytokines played an important role in periodontitis-mediated bone resorption. To examine the effect of selected CpG ODNs on inflammatory factors, we employed ELISA to detect the secretion of TNF-α, IL-1β, IL-6, and IL-17 in RAW264.7 cells. YW002, YW001 and FC004 CpG ODN mainly declined TNF-α level at an early stage (Fig. 7a), and IL-1β decreased at both early and late stages (Fig. 7b). IL-6 and IL-17 were affected at the late stage (Fig. 7c and d), while, IL-6 was dramatically augmented 6 h after stimulation by 2006, YW002, YW001, and FC004 CpG ODN (Fig. 7c). Moreover, YW002 was better in preventing the release of inflammatory factors than 2006 CpG ODN. These three CpG ODNs were immunostimulatory CpG ODNs, which could lead to cytokine secretion by initiating T and B lymphocytes, NK cells, and mononuclear macrophages.^44^ This was the reason for the early augmentation of IL-6 release by these ODNs. During the initial transformation of osteoclast precursor cell RAW264.7 into osteoclasts, intracellular IL-6 expression is up-regulated, which in turn activates the expression of its downstream TNF-α, IL-1β and other cytokines.^45^ We speculate that the possible reason of IL-6 decrease in 24 h is that the expression of IL-6 was stabilized after activating its downstream signals.

Thus, the YW002, YW001, and FC004 CpG ODN could reduce the secretion of inflammatory factors at different stages.

In conclusion, we identified three effective CpG ODNs that can suppress osteoclastogenesis through the OPG/RANKL/RANK pathway, including YW002, YW001 and FC004. These CpG ODNs might be potential ODNs for the prevention and treatment of periodontitis-induced bone absorption.

## Experimental section

### Materials

The 12 ODNs, 2006, *β-actin*, *Nfatc*, *c-fos*, *RANK*, *MMP9*, and FAM labeled ODNs were synthesized by Takara (Dalian, China), and the sequences are listed in ESI Table 1.† The PrimerScript® RT reagent kit and SYBR Green Premix Ex Taq kit were purchased from Takara. The antibodies (cyclin A2, cyclin B1, cyclin D1, cyclin E1, Nfatc, c-fos, RANK, MMP9, OPG, RANKL, β-actin, and horseradish peroxidase-conjugated goat anti-rabbit secondary antibodies) were all purchased from ABclonal (Boston, USA).The RAW264.7 cells (SCSP-5036) were purchased from Cell library of Chinese Academy of Sciences.

### Fluorescence microscopy and laser co-focusing detection

RAW264.7 cells (5 × 10^5^) were seeded in 6-well plates for fluorescence microscopic detection and seeded on the glass slides in 6-well plate at 4 × 10^4^ for laser co-focusing detection. After being incubated for 24 h, the FAM-ODN was added into the wells at 2 μg mL^−1^. After co-culture for 1 h, the cells were washed with PBS, then fixed with 4% paraformaldehyde (Beyotime Biotech Inc., Jiangsu, China) for 10 min. After washing with PBS, the nuclei were stained by DAPI (Beyotime Biotech Inc., Jiangsu, China) for an additional 5 min. After PBS washing, the 6-well plates was directly observed under the fluorescence microscope (Olympus, Osaka, Japan), and the glass slides were fixed by 50% glycerol and detected by laser co-focusing apparatus (FV1000, Olympus, Japan).

### Cell culture and groups

RAW264.7 cells were cultured in growth medium containing L-DMEM (Gibco, USA), 10% fetal bovine serum (BI, Israel), and penicillin (100 U mL^−1^)/streptomycin (100 g mL^−1^) (HyClone Laboratories Inc., Logan, UT). The cells were cultured at 37 °C in 5% CO_2_ incubator (Thermo fisher, Waltham, MA, USA). RAW264.7 cells were divided into 14 groups, cells with PBS as control group, cells with 2006 as positive control group, and cells with ODNs as the experimental groups (12 groups).

### Flow cytometry (FCM) assay

After transfected with FAM-ODN for 1 h, the cells were collected in a dark room, followed by washing and centrifuging three times before transfer into transparent glass test tubes. The indices of flow cytometry (BD bioscience, San Jose, CA, USA) under blue light were set at 488 nm wavelengths. The ratio of the FAM-positive cells/total examined cells was determined quantitatively.

RAW264.7 cells after co-culturing with ODN for 48 h and 72 h were collected to test the G1, S, and G2 phases, and cell apoptosis by cell cycle kit and Annexin V-FITC/PT kit (7sea biotech, Shanghai, China) according to manufacturer's instructions.

### 4,5-Dimethylthiazol-2-yl-2,5-diphenyltetrazolium (MTT) assay

RAW264.7 cells were seeded into a 96-well plate at 4 × 10^3^ cells per well. After 24 h, PBS, 2006, or ODNs were applied, and the working concentration of 2006 and ODN was kept at 2 μg mL^−1^. When co-culturing for 24 h, 48 h, and 72 h, MTT (5 mg mL^−1^ in PBS) (Sigma-Aldrich, St Louis, MO, USA) was added to each well, followed by incubation for an additional 4 h. Then, the MTT solution was removed and DMSO (Sigma-Aldrich, St Louis, MO, USA) was added for 10 min to dissolve the formazan crystals. Absorbance was examined at 492 nm by using a GF-M3000 microplate reader (Shandong, China).

### Live-dead cell staining

RAW264.7 cells were seeded in 6-well plates at 3 × 10^5^ cells per well and were cultured with ODNs for 72 h. After washing with PBS, the live-dead cell staining was conducted using live-dead cell staining kit (Catalog #: K501, Biovision, USA) under dark and at 37 °C for 40 min. The work fluid was discarded and washed with PBS, then live and dead cells were detected under fluorescence microscope (IX71, Osaka Olympus, Japan). The green and red cells represent live and dead cells, respectively.

### Quantitative real-time reverse transcription polymerase chain reaction (qRT-PCR)

RAW264.7 cells were induced to differentiate into osteoclasts by treatment with mouse Receptor Activator of NF-κB Ligand (RANKL) (50 ng mL^−1^, Peprotech, USA) for 2, 4, and 6 days. Then, the total RNAs were isolated using a HiPure Total RNA kit according to the manufacturer's instructions. The mRNAs were investigated with a PrimerScript® RT reagent kit and SYBR Green Premix Ex Taq. The PCR products were evaluated with a MxPro Mx3005P real-time PCR detection system (Agilent Technologies, Santa Clara, CA, USA). The internal control for mRNAs was β-actin. The cycling conditions of mRNAs were as follows: 95 °C for 30 s, followed by 40 cycles of 95 °C for 5 s, 55 °C for 30 s, and 72 °C for 1 min. The 2^−ΔΔ*C*_*t*_^ method was used to calculate the relative expression levels, and the obtained values were averaged from triplicate measurements.

### Western blot analysis

After treatment with ODN for 7 days under mouse RANKL, the proteins from RAW264.7 cells were harvested using RIPA lysis buffer and quantified with bicinchoninic acid protein assay kit (Beyotime Biotech Inc., Jiangsu, China). The proteins were separated on 12% polyacrylamide gels and transferred to polyvinylidene fluoride (PVDF) membranes (Millipore, Billerica, MA, USA). These membranes were blocked with 5% BSA in TBST and incubated with the following primary antibodies: cyclin D1 (A2708), cyclin E1 (A0112), cyclin A2 (A2891), cyclin B1 (A2056), Nfatc (A15339), c-fos (A0236), RANK (A13382), MMP9 (A2095), OPG (A2100), RANKL (A2550), and β-actin (AC026). Horseradish peroxidase-conjugated anti-rabbit secondary antibodies were diluted at 1 : 3000 and incubated at room temperature for 1 h. The signals were detected using an ECL chemiluminescence kit (7Sea biotech, Shanghai, China) with Tanon 5200 (Tianneng, Shanghai, China).

### Tartrate-resistant acid phosphatase (TRAP) and rhodamine phalloidin staining

RAW264.7 cells were seeded in 48-well plates at 1 × 10^4^ cells per well, and cultured in growth medium containing 50 ng mL^−1^ mouse RANKL, and co-cultured with ODN for 4.5 days. Then, the cells were fixed by 4% paraformaldehyde for 25 min under 37 °C. After washing with PBS, the cells were stained by TRAP (Sigma) according to the manufacturer's instructions. The stained cells were permeabilized by 0.5% TritonX-100 for 5 min, and then rhodamine phalloidin work fluid (100 nM, Solarbio, Beijing, China) was added to stain for 30 min under dark and at room temperature. The nuclei were stained by DAPI for an additional 5 min, followed by PBS washing, and then the plate was directly observed under fluorescence microscope.

### Enzyme-linked immunosorbent assay (ELISA)

RAW264.7 cells were seeded into 48-well plates at 5 × 10^5^ cells per well, and co-cultured with ODN for 6 and 24 h. The cells and culture supernatants were collected, followed by centrifugation for 10 min at 3000*g*, and then the supernatants were collected to detect the levels of TNF-α, IL-1β, IL-6, and IL-17 by ELISA kit (Langdun, Shanghai, China) according to the manufacturer's instructions.

### Statistical analysis

The experiments were performed on at least in triplicate. The results are presented as the mean ± standard deviation and were statistically analyzed by Student's *t*-test unless noted otherwise. A two-tailed *P*-value <0.05 was considered statistically significant. *P* values are indicated by *P < 0.05, **P < 0.01, and ***P < 0.001 (similar ^#^*P*), and n.s., non-significant (*P* > 0.05). Statistical analysis was conducted using SPSS version 20.0 (IBM).

## Conclusion

The phosphorothioate CpG ODN could efficiently enter the cells and accumulate in the cytoplasm without vehicle. The selected YW002, YW001, and FC004 CpG ODNs inhibited RAW264.7 cell proliferation by targeting the cyclin proteins to arrest the cells in the G2 phase, and also significantly suppressed osteoclast differentiation through the OPG/RANK/RANKL pathway, as well as the inflammatory cytokine secretion at different stages. The selected CpG ODNs will open a novel avenue for the prevention and treatment of inflammation and bone regeneration in periodontitis.

## Conflicts of interest

There are no conflicts to declare.

## Supplementary Material

RA-010-C9RA11036D-s001
