# Supplementary material for: CpG oligodeoxynucleotides inhibit the proliferation and osteoclastic differentiation of RAW264.7 cells
Source: RSC Adv. 2020 Apr 15;10(25):14885–91. doi: 10.1039/c9ra11036d (PMC9052049; doi:10.1039/c9ra11036d)
Supplement: RA-010-C9RA11036D-s001 [file RA-010-C9RA11036D-s001.pdf]

Electronic Supplementary Material (ESI) for RSC Advances.
This journal is © The Royal Society of Chemistry 2020

Supplementary Table 1 The sequence of CpG OPDs and the primers.

Number	Name	Sequence
	2006	5’-TCGTCGTTTTGTCGTTTTGTCGTT-3’
**1**	FC003	5’-TCTCTCTCTCTCTCTCTCTCTCTC-3’
**2**	SAT05f	5’-CCTCCTCCTCCTCCTCCTCCTCCT-3’
**3**	SAT05d	5’-CTCTCTCTCTCTCTCTCTCTCTCT-3’
**4**	MS19	5’-AAAGAAAGAAAGAAAGAAAGAAAG-3’
**5**	BW001	5’-TCGTCGGGTGCGACGTCGCAGGGGGG-3’
**6**	FC001	5’-TCGGGGACGATCGTCGGGGGG-3’
**7**	FC002	5’-TCGTCGACGTCGTTCGTTCTC-3’
**8**	BW006	5’-TCGACGTTCGTCGTTCGTCGTTC-3’
**9**	YW002	5’-TCGCGAACGTTCGCCGCGTTCGAACGCGG-3’
**10**	YW001	5’-TCGCGACGTTCGCCCGACGTTCGGTA-3’
**11**	FC004	5’-TCGCGAACGTTCGCCCGATCGTCGGTA-3’
**12**	MT01	5’-ACCCCCTCTACCCCCTCTACCCCCTCT-3’
	β-actin	5’-CATCCGTAAAGACCTCTATGCCAAC-3’
		5’-ATGGAGCCACCGATCCACA-3’
	Nfatc	5’-CAAGTCTCACCACAGGGCTCACTA-3’
		5’-TCAGCCGTCCCAATGAACAG-3’
	c-fos	5’-ACGTGGAGCTGAAGGCAGAAC-3’
		5’-AGCCACTGGGCCTAGATGATG-3’
	RANK	5’-GGCTTACCTGCCCAGTCTCATC-3’

	5′-AAGCATCATTGACCCAATTCCAC-3′	
MMP9	5′-GCCCTGGAACTCACACGACA-3′	
	5′-TTGGAAACTCACACGCCAGAAG-3′	
